# Agro-morphological and metabolomics analysis of low nitrogen stress response in *Axonopus compressus*

**DOI:** 10.1093/aobpla/plab022

**Published:** 2021-05-07

**Authors:** Li He, Li Teng, Xiaomin Tang, Wanwan Long, Zhiyong Wang, Yang Wu, Li Liao

**Affiliations:** 1 College of Life Science, Jinggangshan University, Ji’an, Jiangxi 343009, China; 2 Key Laboratory of Genetics and Germplasm Innovation of Tropical Special Forest Trees and Ornamental Plants, Ministry of Education, College of Forestry, Hainan University, Haikou, Hainan 570228, China

**Keywords:** Carpet grass, crop improvement, low nitrogen, secondary metabolism

## Abstract

*Axonopus compressus* also known as carpet grass is a robust, stoloniferous grass that can grow in minimal fertilization and resists well to abiotic and biotic stresses including low nitrogen (LN) stress. This study aimed at characterizing the agro-morphological and metabolome responses to LN in carpet grass leaves. Under LN stress, carpet grass increased yellowness of leaves and root dry matter while reduced turf quality and shoot dry weight. The metabolome comparison between samples from optimum and LN conditions indicated 304 differentially accumulated metabolites (DAMs), which could be classified into 12 major and 31 subclasses. The results revealed that the leaf tissues accumulated more anthocyanins and other flavonoid metabolites under LN stress. Conversely, amino acids, nucleic acids and their derivatives were reduced in response to LN stress. The overall evaluation of individual metabolites and pathways, and previous studies on metabolomes indicated that carpet grass reduced its energy consumption in leaves and increased the level of organic acid metabolism and secondary metabolism in order to resist LN stress conditions.

## Teaser

*Axonopus compressus* (carpet grass) is a robust grass that resists well low nitrogen (LN) stress. Under LN stress, carpet grass increases the root biomass but reduces turf quality and shoot biomass. The metabolome analysis revealed that the leaf tissues accumulate anthocyanins and other flavonoid metabolites under LN stress while amino acids and nucleic acids were reduced. Reduction of energy consumption and increased flavonoid metabolism help carpet grass to resist LN stress.

## Introduction

Nitrogen (N) is an important mineral element required for the processes of plant life activity (Pilbeam 2018). It is an essential element of all nucleic acids which are the building blocks of plant proteins (Lammerts van Bueren and Struik 2017). Nitrogen in soil is usually limited, so soil N deficiency has become an important factor restricting plant growth and development worldwide (Lin *et al.* 2011; Qingfeng *et al.* 2016; Pilbeam 2018). In recent years, to improve the productivity of crops, a large amount of N fertilizer has been applied in agricultural production (Hao *et al.* 2016; Sharma and Bali 2018). The N use efficiency (NUE) for most plant species ranges from 30 to 50 %, while 50 to 70 % of N is lost through denitrification, leaching and volatilization (Ahmed *et al.* 2017; Nguyen and Kant 2018; Sharma and Bali 2018). This loss of N poses a serious threat to the ecological environment, including groundwater pollution and global warming, and threatens human health (Xu *et al.* 2017; Sharma and Bali 2018). Investigating the low-N (LN) tolerance of crops, improving NUE of crops and reducing the amount of N fertilizer applied in agricultural production has become a hot topic (Qingfeng *et al.* 2016; Nguyen and Kant 2018; Zhong *et al.* 2018).

*Axonopus compressus*, also known as carpet grass is a robust, stoloniferous grass with flowering stems that can be up to 45 cm tall. It is widely naturalized and used as a turf and forage in the humid tropics and subtropics (Wang *et al.* 2014; Nawaz *et al.* 2021). The leaves generally form a dense mat that seldom reaches a height of >15 cm (Wang *et al.* 2014). It is often used as a lawn, makes a good ground cover under tree crops and can be used to stabilize the soil. It is one of the most persistent and productive native grasses in plantations and will persist under heavy shade where introduced grasses may not survive (Wang *et al.* 2014). The plant is sometimes harvested from the wild for local medicinal use (Wang *et al.* 2014; Tow *et al.* 2018; Nawaz *et al.* 2021). The whole plant is used as an ingredient in a curative herbal bath and is used to treat heart problems (Wong). It is a useful ground cover and turf in moist, low-fertility soils, particularly in shaded situations. It has been reported for its significant role in bioremediation for soil pollutants (Tow *et al.* 2018). It requires less management and fertilization than most warm season turf grasses (Greene *et al.* 2008). It also provides an excellent erosion control and facilitates reduced herbicide application when used as a soil cover in tree plantations (Wong). It grows well on acidic soils (Baldwin *et al.* 2005) and has excellent tolerance to abiotic and biotic stresses (Wang *et al.* 2014). As soil nitrogen levels decline and under regular defoliation (Lammerts van Bueren and Struik 2017), *A. compressus* can successfully invade pastures composed of high fertility demanding species such as *Paspalum dilatatum*, *Cynodon dactylon* and *Setaria sphacelata*, particularly if shade levels increase (Wong 1990). It is highly tolerant to extensive grazing, and has forage quality higher than *A. fissifolius*. As an abiotic stress-tolerant plant, it can tolerate the minimal nutrient fertilization including N. Various researchers evaluated the effect of abiotic stresses such as soil pollutants (Tow *et al.* 2018) and drought (Nawaz and Wang 2020) on carpet grass; however, our understanding of the LN tolerance in this species is still limited. Hence, it is required to study the mechanism of LN tolerance in carpet grass for the germplasm improvement and breeding for new varieties.

At present, the responses of plants under LN conditions have been studied in depth at the cellular and molecular levels. Using a genome-wide analysis approach, (Zhang *et al.* 2015) identified a specific GATA gene (*GmGATA44*) in soybean that functions against LN stress. Another study used proteomic approaches to investigate how rice proteins respond to LN stress (Ding *et al.* 2011). Various studies in soybean, rice, lotus, maize and other plant species showed that a large number of metabolites responds to LN with a general decrease in amino acids, fatty acids and phytosterols, while an increase of sugars and organic acids was observed (Rasmussen *et al.* 2012; Li *et al.* 2018; Zhao *et al.* 2018; Xin *et al.* 2019; Ganie *et al.* 2020). It has been demonstrated that anthocyanins contribute substantially to the LN tolerance of *Arabidopsis thaliana* based on a metabolomic analysis (Liang and He 2018). The multi-omics method has also been widely used to study plant responses to abiotic stresses. A combination of transcriptome, metabolome and ionome along with hormones and phenotypic analysis has been applied to study changes in the carbon (C), N and phosphorus (P) metabolism in maize source leaves under low-temperature, LN and low-P stresses, and various physiological regulatory processes have been reported (Schlüter *et al.* 2013; Ristova *et al.* 2016).

This study was performed using carpet grass genotype S58 grown in normal N (CK) and LN stress conditions. Here, we compared leaf physiology, and metabolome profiles after 15, 30 and 45 days of exposure to CK and LN conditions. We revealed the physiological differences and differentially accumulated metabolites (DAMs) in LN stress, and annotated the highly enriched molecular pathways in carpet grass for LN tolerance. This research provides a scientific theoretical basis for the improved utilization of high-quality carpet grass resources to cultivate under LN, and it will greatly benefit the improvement of future research not only in carpet grass but also in other commercial crops as well.

## Materials and Methods

### Plant material

The carpet grass *Axonopus compressus*, accession S58 provided by Hainan University, Hainan, China, was used in this study. S58 is an excellent accession with fast propagation, high turf quality, strong drought resistance and aluminium tolerance (Wang *et al.* 2015). The study was conducted in controlled conditions of a greenhouse with temperatures set at 20/8 °C (day/night), a photoperiod of 15 h/9 h (day/night) at the west-gate base of Hainan University.

### Plant growth conditions and sampling

S58 is propagated by vegetative branches in 14-cm-diameter plastic pots containing 2.5 kg washed sand. The healthy seedlings with the same growth were transplanted into the culture medium and fixed with quartz sand. The bottom of each cup was removed and covered with a nylon screen to hold in the sand, but allow roots to grow through it. We suspended cups using 2-cm-thick polyvinylchloride sheets over plastic tanks containing 40 L modified Hoagland solution. The solution components included 5 mM Ca(NO_3_)_2_·4H_2_O, 1.6 mM KNO_3_, 0.5 mM (NH_4_)_2_SO_4_, 1 mM KH_2_PO_4_, 1 mM MgSO_4_·7H_2_O, 5 μM Fe-EDTA, 2.35 μΜ H_3_BO_3_, 0.55 μM MnSO_4_·H_2_O, 0.0385 μM ZnSO_4_·7H_2_O, 0.0165 μM CuSO_4_·5H_2_O and 0.0065 μM H_2_MoO_4_ (Li *et al.* 2018). The pots were divided into CK group and LN stress group, with each group containing 30 pots. In the CK group, plants were cultivated under normal conditions (Hoagland’s nutrient solution with 5.0 mM N concentration). In the LN stress group, seedlings were placed in a modified Hoagland’s solution (0.5 mM N concentration). According to previously adopted method (Li and Chen 2017), potassium sulfate was used instead of potassium nitrate, calcium sulfate instead of calcium nitrate and deionized water was used to prepare Hoagland’s nutrients solution with pH of about 5.5. The electric air pump was continuously ventilated and the nutrient solution was changed every 3 days. We took the leaf samples after 15 (T1), 30 (T2) and 45 (T3) days of LN stress application. For sampling, we randomly selected three pots from each group, took pictures of the plant’ status and leaves were used for the physiological and metabolome analyses.

### Growth parameters

After 45 days of treatment, various growth parameters such as leaf colour, withering rate, turf quality were observed according to reported standards (Liao *et al.* 2011). The harvested grass samples were sterilized at 105 °C and dried to constant weight at 75 °C for 48 h to obtain dry matter weight. The experimental data were analysed using Statistix software including significance analysis and variance analysis.

### Metabolome analyses

#### Sample preparation and extraction.

For the metabolite profiling analysis, the metabolites were extracted from CK- and LN-treated leaf samples (100 ± 5 mg of plant material). The freeze-dried leaf was crushed using a mixer mill (MM 400, Retsch) with a zirconia bead for 1.5 min at 30 Hz. The 100 mg powder was weighted and extracted overnight at 4 °C with 0.6 mL 70 % aqueous methanol. Following centrifugation at 10 000g for 10 min, the extracts were absorbed (CNWBOND Carbon-GCB SPE Cartridge, 250 mg, 3 mL; ANPEL, Shanghai, China, www.anpel.com.cn/cnw) and filtrated (SCAA-104, 0.22 μm pore size; ANPEL, Shanghai, China, http://www.anpel.com.cn/) before UPLC-MS/MS analysis.

#### Detection and analysis of metabolites

##### UPLC conditions.

The sample extracts were analysed using ultra-high-performance liquid chromatography-electrospray ionization-tandem mass spectrometry (UPLC-ESI-MS) (UPLC, Shim-pack UFLC SHIMADZU CBM30A system, www.shimadzu.com.cn/; MS, Applied Biosystems 4500 Q-TRAP, www.appliedbiosystems.com.cn/). The analytical conditions were as follows; UPLC: column, Agilent SB-C18 (1.8 µm, 2.1 mm * 100 mm); the mobile phase was consisted of solvent A, pure water with 0.1 % formic acid, and solvent B, acetonitrile. Sample measurements were performed with a gradient programme that employed the starting conditions of 95 % A, 5 % B. Within 9 min, a linear gradient to 5 % A, 95 % B was programmed, and a composition of 5 % A, 95 % B was kept for 1 min. Subsequently, a composition of 95 % A, 5.0 % B was adjusted within 1.10 min and kept for 2.9 min. The column oven was set to 40 °C; the injection volume was 4 μL. The effluent was alternatively connected to an ESI-triple quadrupole-linear ion trap (Q-TRAP)-MS.

##### ESI-Q-TRAP-MS/MS.

Linear ion trap (LIT) and triple quadrupole (QQQ) scans were acquired on a triple quadrupole-linear ion trap mass spectrometer (Q-TRAP), API 4500 Q-TRAP UPLC/MS/MS system, equipped with an ESI Turbo Ion-Spray interface, operating in positive and negative ion mode and controlled by Analyst 1.6.3 software (AB Sciex). Instrument tuning and mass calibration were performed with 10 and 100 μmol/L polypropylene glycol solutions in QQQ and LIT modes, respectively. Triple quadrupole scans were acquired as metabolic response modifier experiments with collision gas (nitrogen) set to 5 psi. Declustering potential (DP) and collision energy (CE) for individual multiple reactions monitoring (MRM) transitions were done with further DP and CE optimization. A specific set of MRM transitions were monitored for each period according to the metabolites eluted within this period.

### Data processing and analysis

Based on the self-built Metware database (MWDB), according to the secondary spectrum information, the isotopic signals, repeated signals containing K^+^, Na^+^ and NH_4_^+^ ions, as well as the repeated signals of fragment ions with higher molecular weight, were removed during the analysis.

Metabolite quantification was performed by multiple reactions monitoring (MRM) analysis using QQQ mass spectrometry. In MRM mode, the precursor ion (parent ion) of the target material is screened by the four-stage rod to eliminate the corresponding ions of other molecular weight substances to preliminarily eliminate the interference; the precursor ion is induced and ionized by the collision chamber to form a lot of fragment ions, and then the fragment ion is filtered through the triple four-stage rod to select a required characteristic fragment ion to eliminate the interference of non-target ions. It is more accurate and repeatable. After obtaining the mass spectrometry data of metabolites from different samples, the peak area of all mass spectra peaks was integrated, and the peaks of the same metabolite in different samples were integrated and corrected (Fraga *et al.* 2010).

The principal component analysis (PCA) was performed by statistics functions ‘prcomp’ within R (www.r-project.org). The data were unit variance-scaled before unsupervised PCA. Significantly regulated metabolites between groups were determined by variable importance for projection (VIP) ≥ 1 and absolute Log2FC (fold change) ≥ 1. Variable importance for projection values were extracted from orthogonal partial least squares-discriminant analysis (OPLS-DA) result generated using the R package MetaboAnalystR. The data were log transformed (log2) and mean-centred before OPLS-DA. In order to avoid over fitting, a permutation test (with 200 permutations) was performed.

### Kyoto Encyclopaedia of Genes and Genomes functional annotation and enrichment analysis

Identified metabolites were annotated using Kyoto Encyclopaedia of Genes and Genomes (KEGG) compound database (http://www.kegg.jp/kegg/compound/), and mapped to KEGG pathway database (http://www.kegg.jp/kegg/pathway.html). Pathways with significantly regulated metabolites were then fed into metabolite sets enrichment analysis (MSEA). The significance was determined by hypergeometric test’s *P*-values.

## Results

### Growth response of carpet grass to LN

**Figure 1.**
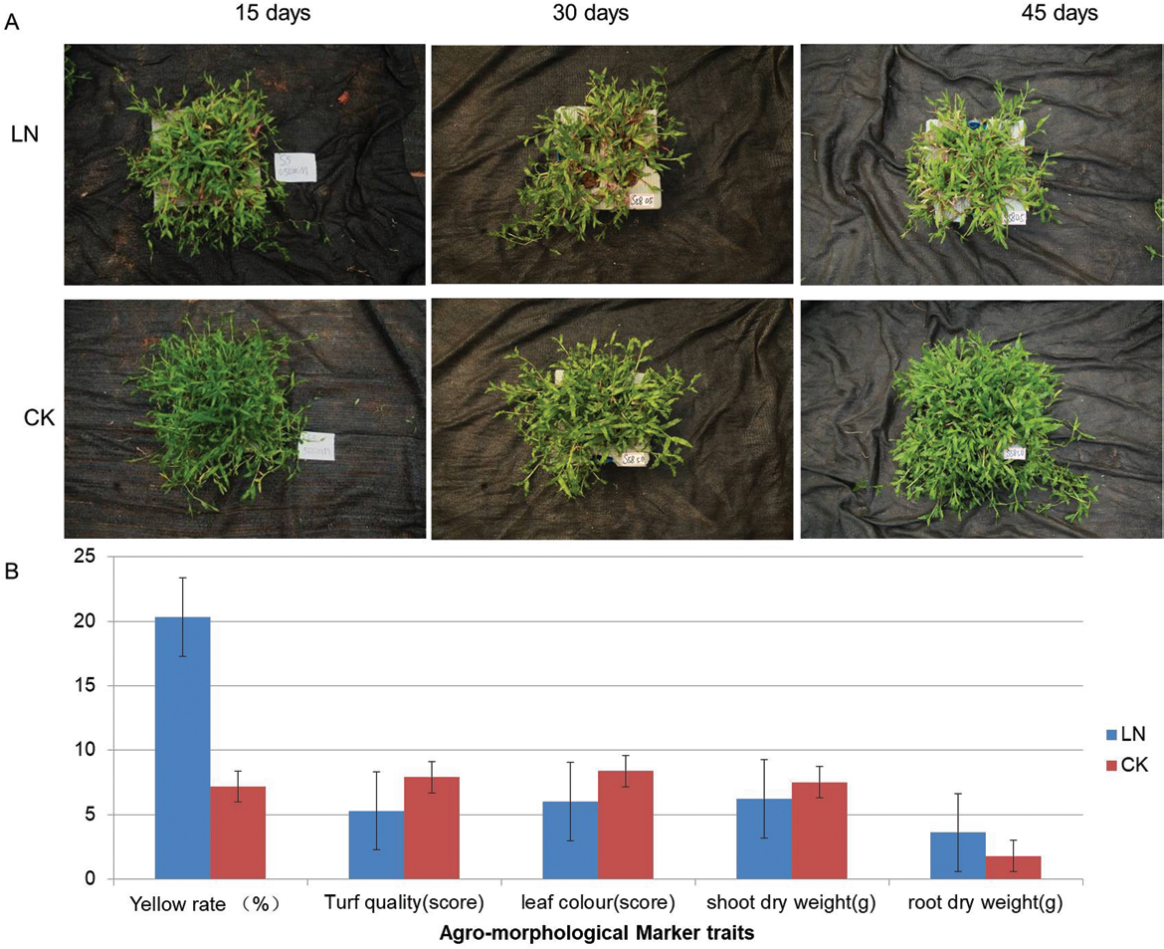
Agro-morphological traits comparison among the sample of carpet grass after 15, 30, and 45 days of LN stress with controlled nitrogen level (CK). (A) Comparison of plant stature, (B) bar graph for agro-morphological traits, error bars indicate mean ± SEs.

Compared with the CK group, LN group revealed significantly affected shoot and root physiology, and clear morphological differences. The yellowness and root dry matter showed a significant increase in LN plants as compared to CK plants. In contrast, turf score, leaf colour scores and shoot dry matter were significantly reduced by LN stress compared to CK group (Fig. 1). The difference in leaf yellowness between CK and LN groups was clearly observable even after 15 days of stress application. Overall, our results showed strong effects of LN stress on carpet grass growth, and biomass accumulation.

### Metabolite profiling in carpet grass under CK and LN treatments

The metabolome profiles in the leaves of the carpet grass genotype S58 were analysed using ultra-high-performance liquid chromatography-electrospray ionization-tandem mass spectrometry (UPLC-ESI-MS/MS system). A total of 586 metabolites were detected in carpet grass leaves, belonging to 12 major and 31 subclasses as summarized in Table 1 and **Supporting Information—Table S1**. Among the 12 major classes, 147 metabolites were flavonoids, followed by phenolic acids (74), lipids (69), amino acid derivatives (65), nucleotides and derivatives (51), organic acids (49), alkaloids (33), lignans and coumarins (25) and terpenoids (7). Quinones and tannins along with others 63 metabolites were less represented.

**Table 1.** Various types and classification of various metabolites in carpet grass.

Sr. no.	Classes of metabolites		Metabolites			
	Major classes	Subclasses	All	Conserved	Differentially accumulated	Associated to LN stress
1	Alkaloids	Alkaloids	18	12	6	1
2		Phenolamine	8	3	5	
3		Plumerane	6	2	4	1
4		Quinoline alkaloids	1	0	1	
5	Amino acids and derivatives	Amino acids and derivatives	65	23	42	4
6	Flavonoids	Anthocyanins	9	0	9	6
7		Dihydroflavone	5	1	4	1
8		Dihydroflavonol	3	0	3	1
9		Flavonoid	53	11	42	9
10		Flavonoid carbonoside	10	5	5	1
11		Flavonols	57	4	53	34
12		Isoflavones	10	4	6	4
13	Lignans and coumarins	Coumarins	8	4	4	
14		Lignans	17	13	4	
15	Lipids	Free fatty acids	33	27	6	
16		Glycerol ester	14	10	4	
17		Lysophosphatidylcholines	12	12		
18		Lysophosphatidylethanolamine	10	9	1	
19	Nucleotides and derivatives	Nucleotides and derivatives	51	20	31	2
20	Organic acids	Organic acids	49	39	10	3
21	Others	Others	8	5	3	1
22		Saccharides and alcohols	39	13	26	15
23		Stilbene	2	2		
24		Vitamin	14	11	3	1
25	Phenolic acids	Phenolic acids	74	46	28	4
26	Quinones	PhenAnthraquinones	1	1		
27	Tannins	Tannin	2	1	1	
28	Terpenoids	Monoterpenoids	1	1		
29		Sesquiterpenoids	2	1	1	
30		Terpene	2	2		
31		Triterpene	2	0	2	
Total		Total	586	282	304	88

**Figure 2.**
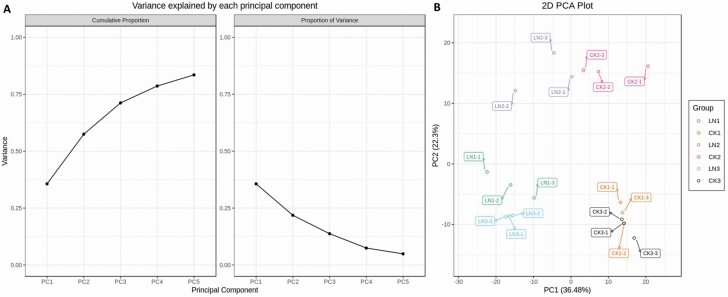
Principal component analysis (PCA) of samples at various sampling time as 15 days (1), 30 days (2) and 45 days (3) after exposure to LN stress and optimum nitrogen (CK) in three replications. (A) Variance explained by PCA; (B) loading plot for distribution of samples on the basis of PCA.

The general influence of LN stress on the metabolite profile of carpet grass leaves was first tested by PCA. The dispersion between quality control (QC) samples showed that the metabolic analysis instrument had stable and reliable data detection and could thus be used for subsequent analysis. To compare the samples distribution pattern, a scatter plot was drawn on the basis of PCA scores. There was an obvious separation between samples within the CK and LN stress groups (Fig. 2). It revealed the dependability of metabolite detection and the metabolomics analysis. The first principal component (PC1) explained 36.48 % of the total variance in leaf metabolome among samples, which separated the CK and LN stress groups (Fig. 2B), indicating that the LN stress had a substantial effect on metabolite concentration. PC2 differentiated between samples at different collection times T1, T2 and T3 (15, 30 and 45 days, respectively). It grouped the T1 and T3 samples against the T2 samples, and explained 22.3 % of the total variation in leaf metabolome among samples (Fig. 2B). The closer clustering of biological replicates for T1 and T3 than T2 also revealed the higher sampled quality in T1 and T3 while relatively higher variation among T2 samples.

### DAMs under LN treatment

**Figure 3.**
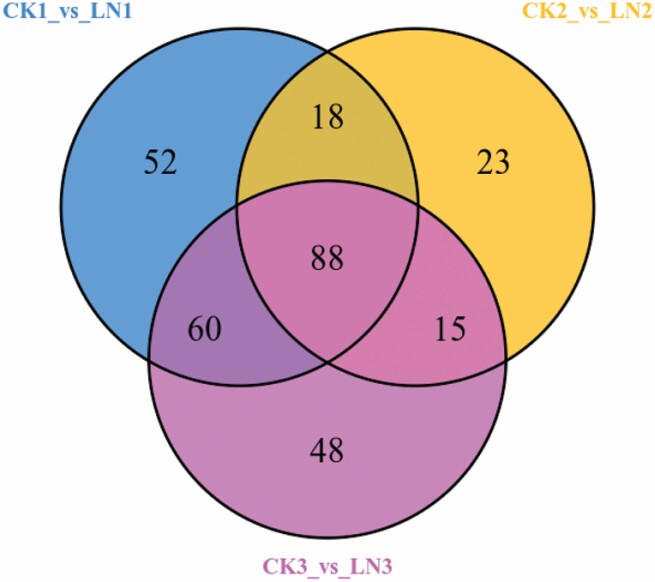
Venn diagram of comparative analysis among samples for DAMs.

The OPLS-DA was performed on the metabolic profiles, and significantly DAMs were selected with VIP > 1. According to the above criterion, a total of 304 DAMs (51.87 % of total metabolites) were detected in carpet grass leaves as affected by LN stress **[see****Supporting Information—Table S1****]**. As compared to the control treatment, 218, 144 and 211 DAMs were obtained at T1, T2 and T3 between CK and LN, respectively. Among these metabolites 157, 103 and 154 were up-accumulated, while 61, 41 and 58 metabolites were down-accumulated at T1, T2 and T3 respectively. Among the total 304 DAMs, 88 were differentially accumulated at all stages, representing the core metabolome responsive to LN independently of the stress duration. In addition, 93 were differentially accumulated among two of three stages, while 123 metabolites showed time-specific accumulation. Among the 123 sampling time-specific DAMs, 52, 23 and 48 were specifically observed at T1, T2 and T3, respectively (Fig. 3). Overall metabolite concentrations were increased with LN stress.

Among the DAM, most of them (40 %) were ‘flavonoids’ followed by ‘amino acids and derivatives’ (13.8 %), ‘nucleic acids and derivatives’ (10.2 %) and ‘phenolic acids’ (9.2 %) (Table 1). Among the core-conserved 88 DAMs responsive to LN stress at each growth stage **[see****Supporting Information—Table S1****]**, seven metabolites including one ‘plumerane’ (tryptamine), one ‘alkaloid’ (gramine), four ‘amino acid derivatives’ (*N*,*N*-dimethylglycine, L-aspartic acid, L-tyramine and L-tryptophan) and one ‘nucleotide derivative’ (8-hydroxyguanosine) were down-accumulated, while the remaining 81 metabolites were up-accumulated in response to the LN stress.

**Figure 4.**
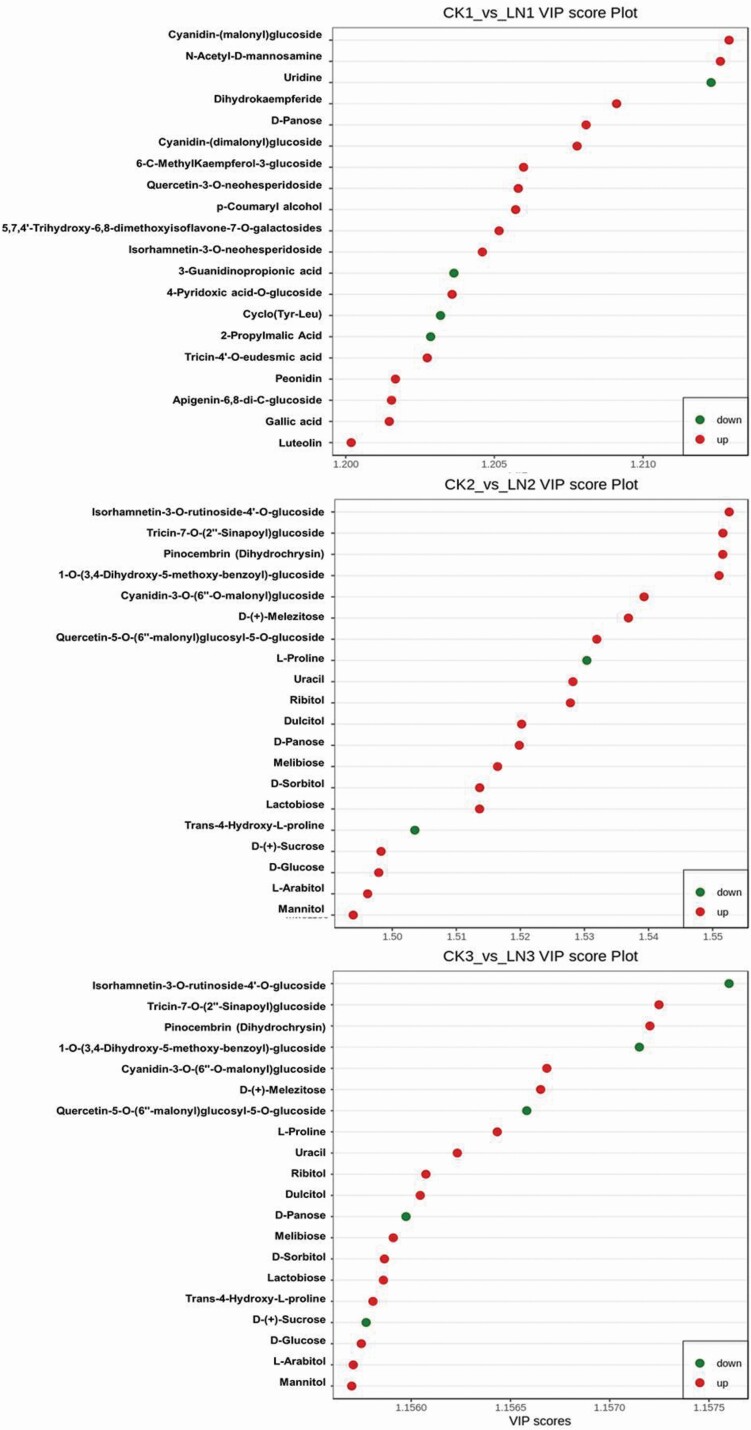
Variable importance for projection (VIP) scores analysis based on the weighted coefficients of the OPLS-DA, for control (CK)- versus LN-stressed samples after 15 (CK1 vs LN1), 30 (CK2 vs LN2) and 45 (CK3 vs LN3) days after LN stress application.

For individual metabolites, VIP scores were used to rank the contribution of metabolites to the discrimination between CK and LN groups, which are based on the weighed coefficients of the OPLS-DA model (Fig. 4). Most notably in T3, large accumulations of saccharide D-sedoheptulose 7-phosphate (15.47 Log2fold upregulation), nucleotide derivative 2′-deoxyadenosine-5′-monophosphate (14.05 Log2fold upregulation) and flavonoid quercetin-5-*O*-(6″-malonyl)glucosyl-5-*O*-glucoside (13.19 Log2fold upregulation), while reduced accumulation of triterpene 24,30-dihydroxy-12(13)-enolupinol (13.94 Log2fold downregulation) was observed.

### KEGG pathway enrichment analysis

**Figure 5.**
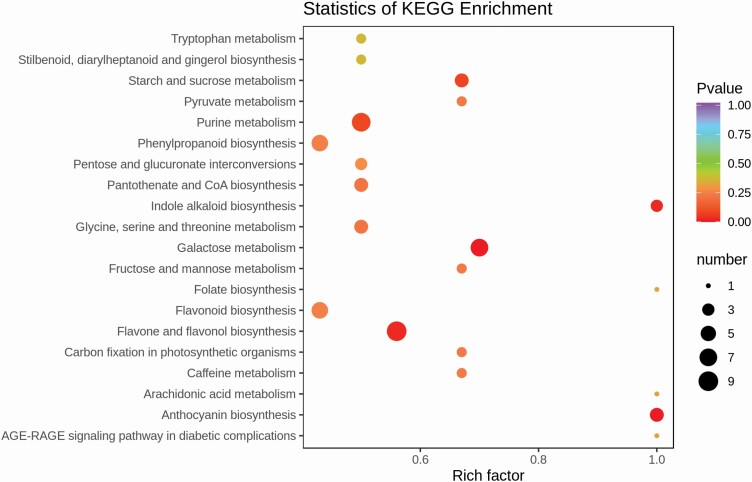
Metabolome enrichment analysis for among the top 20 annotated KEGG pathways.

The DAMs in T1, T2 and T3 were annotated in 62, 66 and 66 KEGG pathways, respectively. Overall, 76 pathways were discovered by pairwise comparison among the samples of CK and LN groups, of which 52 pathways were found to be involved regardless of sampling time. Among the annotated pathways, two pathways including ‘metabolic pathways’, and ‘biosynthesis of secondary metabolites’, pathways were observed to be enriched with maximum metabolite frequency of 81 % and 46 %, respectively, and significance of DAMs **[see****Supporting Information—Table S2**; **Supporting Information—Fig. S1****]**. Generally, metabolites involved in these two pathways were up-accumulated under LN stress (Fig. 5; **see****Supporting Information—Table S2**).

We also evaluated the rich factor (RF) among different pathways that is the ratio of the number of DAMs in the corresponding pathway to the total number of metabolites detected by the pathway, and extracted the top 20 enriched pathways. Among them, ‘anthocyanin biosynthesis’ showed the highest enrichment score and indicated its affinity to respond the LN stress (Fig. 5).

## Discussion

In plant leaf, the N metabolism is closely related to life activities, such as photosynthesis and metabolite biosynthesis. As a centre of photosynthesis, leaves are vulnerable under LN conditions because they have direct effect on plant growth and development with minor to diverse effects from early to late growth stages (Li *et al.* 2009). The plants of carpet grass grown in LN stress were observed to have an increased yellowness and have significant difference among the samples from CK for turf quality, leaf colour scale, and shoot and root dry weights, which is consistent with previous studies (Stewart *et al.* 2001). It is well known that the nitrogen application has a significant positive correlation with turf grass colour and turf quality. Various studies have reported the reduced leaf area index and above-ground biomass (Tolley and Mohammadi 2020), while increased root dry weight (Liu *et al.* 2020) in LN stress. The decreased shoot dry weight and increased root dry weight under LN stress indicated the significant response of carpet grass to LN stress. Therefore, this study focused on the mechanisms of LN response associated with plant metabolism.

The DAMs in carpet grass under CK and LN stress conditions belonged to 12 major and 31 subclasses (Table 1). The most abundant (40 %) DAMs were flavonoids. Nitrogen limitation promoted increase in flavonoids, while reduced amino acids, and nucleic acids contents. The carpet grass leaf tissue was found to contain 122 flavonoids related DAMs which all were found to increase in response to reduced N availability. Flavonoids are a large family of polyphenolic compounds found in plant tissues (Haslam 1998). Flavonoids mainly occur as sugar conjugates and are concentrated in the upper epidermis of leaves and skins of fruits (Stewart *et al.* 2001). Limiting N availability also induced higher concentrations of flavonols in leaf tissues. A wide range of functions have been proposed for flavonoids in relation to abiotic stresses and a variety of nutrient deficiencies in plants are characterized by an accumulation of flavonoids, notably the coloured anthocyanin. The carpet grass grown in CK condition showed greenish, while yellowish in LN stress indicating an increase in anthocyanin contents. The metabolome analysis showed the inverse association of flavonoid contents to nitrogen in carpet grass that is in accordance with previous study on metabolomes of *Arabidopsis* seedlings (Stewart *et al.* 2001). It has been reported that the anthocyanin content can increase by 3·4-fold in response to N stress (Bongue-Bartelsman and Phillips 1995).

It was also found that nitrogen deprivation greatly increased the levels of chalcone synthase (CHS) and dihydroflavonol reductase (DFR) mRNA. An earlier study with apple demonstrated an increased accumulation of phenylalanine ammonia lyase (PAL) following reduced availability of nitrogen and potassium (Stewart *et al.* 2001). One explanation for increased flavonoid synthesis under nitrogen stress is that enhanced PAL activity will release nitrogen for amino acid metabolism, whereas the C products are shunted via 4-coumaroyl-CoA into the flavonoid biosynthetic pathway (Stewart *et al.* 2001). Alternatively, nitrogen limitation will affect photosynthesis by decreasing available chlorophyll and disrupting photosynthetic membranes due to starch accumulation. This may lead to increased sensitivity to high light levels. The production of photoprotective pigments such as anthocyanin and flavonols may afford protection against light-induced oxidative damage (Stewart *et al.* 2001). Flavonols are known to accumulate in the skins of tomato fruits (Stewart *et al.* 2001) and could therefore filter out damaging wavelengths of radiation.

In this study, LN stress caused strong shifts in the leaf metabolome. Lower levels of amino acids accumulate after LN stress in *Arabidopsis* and maize (Amiour *et al.* 2012; Schlüter *et al.* 2012). Previously, the glycolysis and tricarboxylic acid metabolism pathways were observed to be enriched at initial (tillering) stage of plant growth in rice, while the nitrogen and proline metabolism were enriched at later (booting) stage (Zhao *et al.* 2018). The accumulation of energy-saving amino acids may be beneficial to the plant growth, contributing to its higher LN tolerance (Quan *et al.* 2016). Under LN stress, the amino acid and derivative contents, including 42 metabolites (Table 1), showed downward trend, indicating an inhibited amino acid metabolism. Thus, the carpet grass plants just like to soybean resist LN stress by reducing energy consumption (Liu *et al.* 2020). The proline accumulation was also reduced by LN stress that could alleviate the stress and improve plant resistance (Sharma and Dietz 2006). Similarly in barley, LN stress induced the tissue-specific changes in C and N partitioning, and the patterns of energy-saving, amino acid accumulation and C distribution in favour of root growth that contribute to its higher LN tolerance (Quan *et al.* 2016).

We found five organic acids as malic acid, threonic acid, urocanic acid, dihydrobenzoic acid and shikimic acid significantly up-accumulated in response to LN stress in carpet grass (Table 1; **see****Supporting Information—Table S1**). The accumulation of organic acids improves plants adaptation to poor environments (Liu *et al.* 2020), and plays an important role in maintaining the intracellular ion balance to adapt to alkaline stress (Gong *et al.* 2014). The secondary metabolites biosynthesis pathway was observed to be the most enriched pathway under LN stress. In *Arabidopsis* leaves, tomato leaves and maize leaves, N deficiencies lead to increase in carbohydrates, sugars and secondary metabolites (Schlüter *et al.* 2012). Among 26 sugar alcohol DAMs, four were significantly reduced at one of the three sampling times, while the rest showed increasing trend. These accumulations also contribute towards the structural development and LN tolerance. Secondary metabolites are products that adapted to the environment during the long-term evolution of plants, and their recognized ecological functions include disease, insect and environmental stress resistance (Li *et al.* 2017; Liu *et al.* 2020). The secondary metabolites such as shikimic acid are associated with the shikimate metabolic pathway. The shikimic acid pathway in plants is the main bridge between glucose metabolism and secondary metabolism, and it is the main synthetic pathway of aromatic amino acids, plant hormones and a variety of important active secondary metabolites (Liu *et al.* 2020). This pathway plays a very important role in plant growth and development, signal transduction, disease resistance and abiotic stress tolerance in plants (Becerra-Moreno *et al.* 2015). For example, a study evaluated the LN stress effect in soybean and revealed a high accumulation of metabolites related to the shikimic acid pathway such as carotenoids, soluble sugars, organic acids under stress (Li *et al.* 2018).

It can be summarized that carpet grass reduced its energy consumption and increased the level of organic acid metabolism and secondary metabolism in leaves under LN stress as compared to CK conditions to develop the LN tolerance (Liu *et al.* 2020).

## Conclusions

*Axonopus compressus* can grow in minimal fertilization and resists well to abiotic and biotic stresses including LN stress. The agro-morphological and metabolome responses to LN in carpet grass leaves were observed. Under LN stress, carpet grass increased yellowness of leaves and root dry matter while reduced turf quality and shoot dry weight. The comparative metabolome analysis revealed the 304 DAMs. More anthocyanins and other flavonoid metabolites were accumulated in leaf tissues under LN stress while amino acids, nucleic acids and their derivatives were reduced in response to LN stress. Individual metabolite and pathway analysis indicated the increased level of organic acid metabolism and secondary metabolism under LN stress conditions.

## Supporting Information

The following additional information is available in the online version of this article—

**Figure S1.** Metabolome enrichment analysis based on bar graph among the annotated Kyoto Encyclopaedia of Genes and Genomes (KEGG) pathways.

**Table S1.** Metabolome profile in carpet grass samples under normal (CK) low nitrogen (LN) stress conditions.

**Table S2.** List of annotated Kyoto Encyclopaedia of Genes and Genomes (KEGG) pathways and their enrichment among the differentially accumulated metabolomes in carpet grass after 15 days (T1), 30 days (T2) and 45 days (T3) after exposure to stress.

plab022_suppl_Supplementary_Figure_S1

plab022_suppl_Supplementary_Table_S1

plab022_suppl_Supplementary_Table_S2

## Notes

**Phenome, Genome & Environment. Chief Editor:** Colleen Doherty
