# Supplementary figures and images for: Agro-morphological and metabolomics analysis of low nitrogen stress response in Axonopus compressus
Source: AoB Plants. 2021 May 7;13(4):plab022. doi: 10.1093/aobpla/plab022 (PMC8256886; doi:10.1093/aobpla/plab022)

KEGG Classification

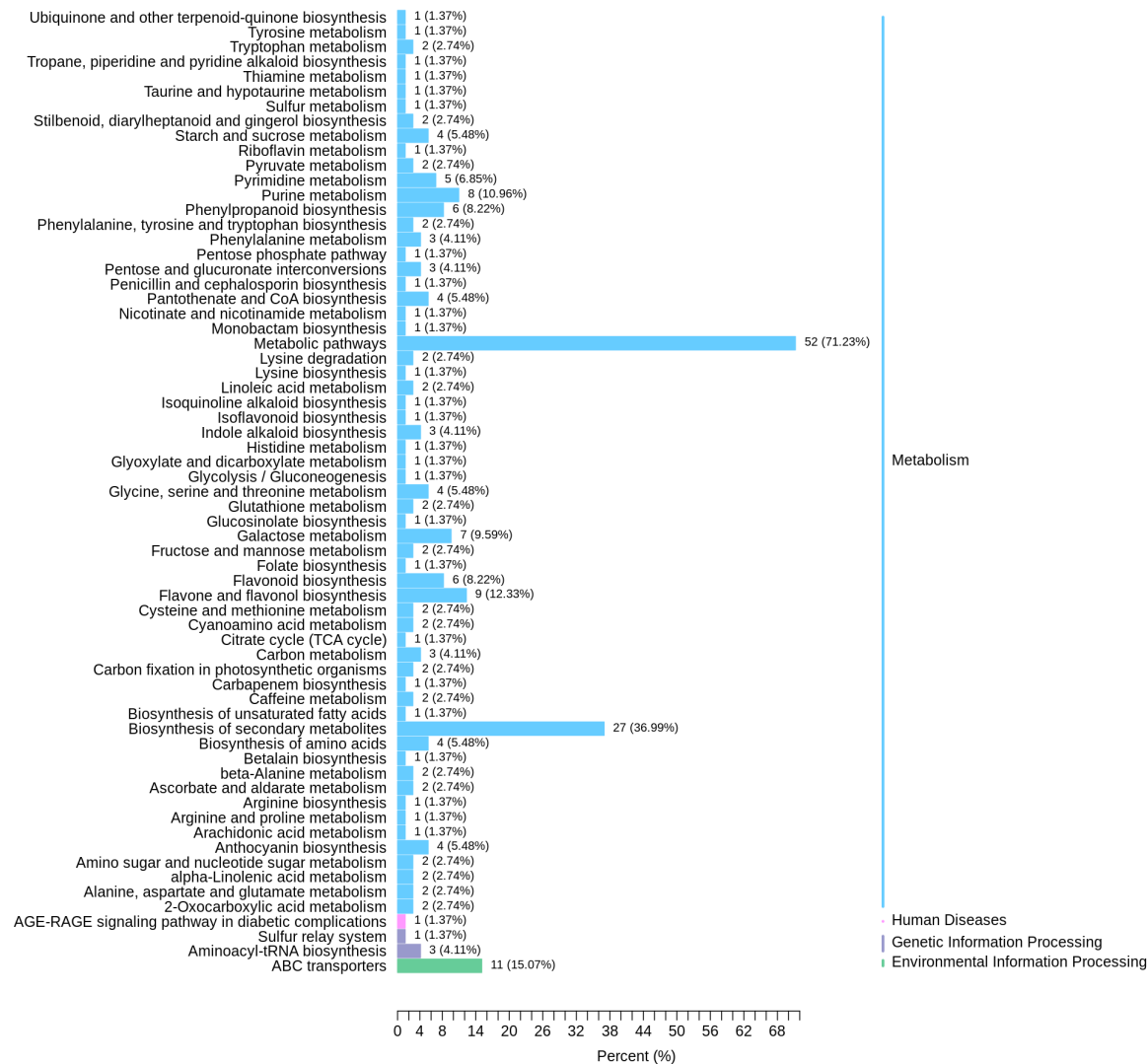

Supplement: plab022_suppl_Supplementary_Figure_S1 [file plab022_suppl_supplementary_figure_s1.pdf]
